# A comparison of classroom and online asynchronous problem-based learning for students undertaking statistics training as part of a Public Health Masters degree

**DOI:** 10.1007/s10459-012-9368-x

**Published:** 2012-04-03

**Authors:** N. de Jong, D. M. L. Verstegen, F. E. S. Tan, S. J. O’Connor

**Affiliations:** 1 Department of Health Services Research, CAPHRI School for Public Health and Primary Care, Faculty of Health, Medicine, and Life Sciences, Maastricht University, P.O. Box 616, 6200 MD Maastricht, The Netherlands; 2 Department of Educational Development and Research, Faculty of Health, Medicine and Life Sciences, Maastricht University, Maastricht, The Netherlands; 3 Department of Methodology and Statistics, Faculty of Health, Medicine and Life Sciences, Maastricht University, Maastricht, The Netherlands; 4 Department of Acute Care, Faculty of Health Care and Social Sciences, University of Bedfordshire, Bedfordshire, UK

**Keywords:** Blended learning, Online asynchronous learning, Problem-based learning, Public health, Statistics training

## Abstract

This case-study compared traditional, face-to-face classroom-based teaching with asynchronous online learning and teaching methods in two sets of students undertaking a problem-based learning module in the multilevel and exploratory factor analysis of longitudinal data as part of a Masters degree in Public Health at Maastricht University. Students were allocated to one of the two study variants on the basis of their enrolment status as full-time or part-time students. Full-time students (n = 11) followed the classroom-based variant and part-time students (n = 12) followed the online asynchronous variant which included video recorded lectures and a series of asynchronous online group or individual SPSS activities with synchronous tutor feedback. A validated student motivation questionnaire was administered to both groups of students at the start of the study and a second questionnaire was administered at the end of the module. This elicited data about student satisfaction with the module content, teaching and learning methods, and tutor feedback. The module coordinator and problem-based learning tutor were also interviewed about their experience of delivering the experimental online variant and asked to evaluate its success in relation to student attainment of the module’s learning outcomes. Student examination results were also compared between the two groups. Asynchronous online teaching and learning methods proved to be an acceptable alternative to classroom-based teaching for both students and staff. Educational outcomes were similar for both groups, but importantly, there was no evidence that the asynchronous online delivery of module content disadvantaged part-time students in comparison to their full-time counterparts.

## Introduction

### Background and context for the study

This paper outlines the results of a case-study designed to compare outcomes from a traditional classroom-based, face-to-face statistics module for students undertaking a Public Health Masters degree with a parallel asynchronous online variant delivered at the same point during the academic year. A classroom-based module on the multilevel analysis of longitudinal data and exploratory factor analysis had previously been taught as part of the MSc Public Health degree at Maastricht University but unlike other modules delivered within the University, this had not been taught using problem-based learning and teaching approaches on the basis that the content was considered too declarative in nature. This is not unusual however. Bland (2004) for instance, points out that problem-based learning has not been used extensively for the teaching of statistics content within the biomedical sciences for much the same reason. As a consequence, the module had historically been taught using traditional didactic teaching methods—primarily in the form of face-to-face lectures and practical exercises using the SPSS software package. However, in view of advances in our knowledge about the cognitive and motivational effect of problem-based learning in small groups (Dolmans and Schmidt 2006), it was decided to develop a problem-based module using Maastricht University’s ‘seven-step’ approach, namely: clarifying and agreeing working definitions, terms and basic concepts, defining the problem, analysing and restructuring the problem, formulating learning goals, and individual study followed by group feedback on the learning attained. In particular, it was believed that input from a problem-based learning group tutor who was also a core member of the statistics teaching team would help students synthesize the many difficult concepts addressed within the module and help them to consolidate learning acquired from more didactic components such as lectures.

A problem-based module was developed according to the university’s standard module development procedures by those responsible for statistics education within the MSc Public Health programme. It was quickly recognised, however, that part-time students in full-time employment might experience difficulty attending problem-based learning tutorials in addition to other compulsory modules during their limited study time. In response to student feedback on the proposals, it was decided to develop and pilot an online variant of the new module which could be undertaken independently by part-time students in full-time employment in order to better accommodate their work, study and family commitments. The module coordinator (FT) and others responsible for the development of the statistics module met with the Faculty’s designated blended learning coordinator (NdJ) and an advisor from the Faculty’s Institute of Education (DV) to develop an online version of the module. During the course of four meetings, the module content was reviewed and the problems adapted slightly so that they better suited the online learning environment. Notwithstanding differences in the means of its delivery, the espoused content of both module variants remained exactly the same, with students accessing the same reading materials and lectures (either face to face within the classroom setting or via a recording of the same lecture in the online variant). However, given the differential nature of the part-time students’ work commitments, it was decided that the online variant would be delivered asynchronously for the most part, allowing them to access the materials and contribute to PBL activities at the time which best suited them. The sample size for the unmatched cohort case-study was intentionally restricted to two groups tutored by the same member of the module teaching team in order to reduce ‘teacher effect’ as a possible source of bias.

### Problem-based learning

The growth in popularity of problem-based learning and teaching methods is based in large part upon an improved understanding of the ways in which students learn, and relatively recent insight into the importance of student-centered learning. Within this context, problem-based learning is based on four primary principles namely: it should be constructive, it should be self-directed, it should be collaborative, and it should be contextual (Dolmans et al. 2005). An important aspect of the approach is that the student is viewed as an active participant in their learning rather than a passive recipient of knowledge (Dolmans et al. 2005; Dolmans and Schmidt 2006; Mok 2009). In addition, it affirms that learning is a socially constructed or ‘communal’ activity which is expansive in nature (Engeström and Sannino 2010). This enables participants to draw upon prior knowledge or experience (including that derived from the world of work) as the starting point for their ongoing learning and development and is a highly appropriate strategy for adult education (Engeström 2001). It is particularly suited to the continuous professional development of healthcare professionals since it builds upon an existing repertoire of experience and takes them through a process of ‘collective concept formation’ which enables them to better understand the polycontextual world in which they live and work (Engeström et al. 1995, p. 321).

Within this context, and for the purposes of the study, problem-based learning was conceptualised as a collaborative process in which each of the following conditions are met: participants have a common goal or objective for their learning, they share responsibility for that learning, are mutually dependent on each other for their learning needs, and are able to agree upon a course of action intended to meet those needs through open dialogue and interaction (Dolmans et al. 2005). Furthermore, it was hypothesized that learning which is situated within meaningful contexts, cases or problems better facilitates knowledge transfer and nurtures the ability of learners to solve real-life problems whilst fostering communication, negotiation and cooperation skills which are highly valued by (potential) employers (Woltering et al. 2009; Suzuki et al. 2007; Cunningham et al. 2006). It has also been suggested that when aligned with online or distance learning teaching strategies, students also develop important technical competencies such as internet searching, computer literacy and self-study skills in addition to an enhanced sense of self-efficacy as a result of their online learning (Guy and Lownes-Jackson 2010).

### Online learning

Any educational intervention making use of the internet (or a local intranet) can be described as online or web-based learning (Goodrich 2007). In addition to the development of self-efficacy and computer literacy skills, the advantages of this learning and teaching approach include overcoming barriers of time and distance, economies of scale, greater student centeredness, and the ability to introduce novel instructional methods to individual modules, courses or programmes of study (Turney et al. 2009). However, social isolation, considerable up-front development costs, and occasional technical problems are identified in the literature as disadvantages to this approach, particularly where the principles of effective learning are not incorporated into the initial programme design (Cook 2007). Mayer (2009) argues that the ready availability of technology is not adequate reason for this to be employed on each and every occasion, thus an empirically based understanding of how people learn and how they react to online learning is also necessary when considering the introduction of such an approach. Online learning should be learner-centered and begin with an understanding of how the human mind works. As such, it should always focus upon the primary *learning* task (Mayer 2009). Decisions about the structure and focus of online modules or courses should therefore be based on the kind of learning desired, the optimum timeframe in which learning is to take place, and ways in which learning can be achieved in the most effective manner from both a cost and student centered perspective (Cook 2007). Sitzmann et al. (2006) argue that online learning represents a non-linear instructional medium that may encourage deeper processing and cognitive flexibility, but note that most studies field have focused on declarative knowledge rather than procedural knowledge or training reactions. It was hypothesized therefore, that the combination of online and problem-based learning and teaching methods in the variant reported upon here would demonstrate important synergies from both staff and student perspectives in addition to any timetabling issues that it would help to overcome. It was hoped that the addition of problem-based learning would strengthen ‘learner control’, one very important requirement for the success of online teaching approaches and return the focus of the module to the practical statistical problems thus addressed rather than the software per se. Learner control refers to the extent to which learners ‘have control over their learning experience by affecting the content, sequence or pace of material (Sitzmann et al. 2006, p. 631), all of which are encouraged through the more student-centered problem-based learning approach.

### The synergy of online and problem-based learning

Suzuki et al. (2007) reported that online problem-based learning offers many advantages to adult learners. It enables communication between students wherever they may be and facilitates learning on a global scale which promotes not only the acquisition of knowledge and computer literacy, but also an understanding of different social and cultural perspectives within international learning environments. Whilst this was not a primary outcome measure in our study, it was hypothesized from the literature that in tandem, the two approaches might facilitate greater multidisciplinary learning and nurture an improved sense of teamwork when employed within the multidisciplinary context. This makes the use of online problem-based learning programmes particularly salient for healthcare professionals who may, by virtue of differing working patterns or time commitments find it difficult to engage in face-to-face learning with others on a regular basis. Moreover, the adaptation of problem-based learning for online delivery offers the opportunity to use new teaching methods and diverse learning foci which, when incorporated into an online educational strategy offer advantages for both the students and those who teach them (Gray and Tobin 2010; Campbell et al. 2008; Sancho et al. 2006; Riffell and Sibley 2005; Garrison and Kanuka 2004). Savin-Baden (2007) however, argues that different approaches to face-to-face problem-based learning can both guide and inform the way that problem-based learning is applied to online settings, although it is likely that higher levels of student motivation and self direction will be called for in such settings if improvements in self-efficacy are to be achieved (Guy and Lownes-Jackson 2010). This is essential as learning undertaken in this way is both linear-temporal and socio-spatial (Engeström 2008). Another complicating factor is that problem-based learning is unlikely to be suitable for every online module, course or educational programme since these are far from homogenous and online education itself has been interpreted in many different ways. This makes it difficult to draw firm conclusions with respect to its functionality since many factors may play a role in this. These include the background, preferences, motivation and time-schedules of students, curriculum design and content, differences in the capacities and preferences of teachers, and variations in the teaching resource available for teaching using these methods (Cook 2007), all of which were considered in the development of our module.

### Description of the classroom-based and online module variants

Both module variants lasted 8 weeks. Weekly tasks for the tutorials consisted of statistical PBL problems and a variety of questions which the students had to answer in collaboration with other students in their group. One week was allocated for the completion of each problem-based learning task in both the online and classroom-based variants. Similarly, both sets of students used the localized version of Blackboard 8.1 (EleUM) to access reading materials, module handbooks and information in relation to the PBL tasks etc., including access to the online discussion board facility ‘Polaris’. Students undertaking the online variant met face-to-face with their fellow students, the PBL tutor and the blended learning coordinator in week 1 of the module in order to orientate students to the online variant and to each other. Research suggests that initial meetings at the start of an online programme help to foster and strengthen commitment to each other and the completion of the required learning tasks in addition to the development of ‘cognitive presence’ (Vaughan and Garrison 2005). Other than this, students on the online module variant did not meet each other face to face again until the final week of the module (week 8) when attendance at the University was required for a compulsory unseen examination. We have chosen not to use the phrase ‘blended learning’ for this module and the study which accompanied it since the vast majority of the declarative or espoused knowledge, and all of the procedural knowledge or practical training took place in the online learning environment and not in the face-to-face meetings, the purpose of which were largely social, administrative or preparatory in nature (Sitzmann et al. 2006).

Eleven full-time students followed the classroom-based variant of the module which was delivered 1 day per week. This consisted of a mixture of classroom-based lectures, guided practice using the SPSS software package in a computer ‘laboratory’, and classroom-based PBL tutorials. Twelve part-time students participated in the online variant which included the asynchronous delivery of video recorded lectures and a series of individual SPSS activities designed to mirror the guided practice given to full-time students within the classroom. Classroom-based PBL tutorials were replaced by an asynchronous discussion board on Polaris. Here, students discussed the same tasks as those addressed by the full-time classroom based students, but these were facilitated by the students themselves rather than the PBL tutor on a revolving basis. The ‘responsibility’ principle was used to make sure that each of the online students engaged in the learning activities and contributed fully in the module in their turn (Gray and Tobin 2010; Barnard et al. 2009). This was operationalised by a different group of 2–4 students taking responsibility for each weekly PBL task. They were asked to provide a first answer to the questions within 3–4 days of the discussion board being opened and after this period, the other students were invited to join the discussion and contribute their own thoughts or suggestions as to the problem which had been posed. The other students could post messages and/or attachments during this period, and others could indicate their agreement of disagreement with these contributions which could be seen by each of the other students. Those responsible for the weekly task were then required to summarise their own and their colleagues’ solutions to the problem and send this to the tutor by a predetermined deadline. The tutor then reviewed the summary document and recorded audiovisual feedback which was then delivered to the students via Blackboard (ELeUM). If necessary, illustrations or additional reading were also added to further explain the statistical concepts under discussion.

### The role of the blended learning coordinator in the module

The blended learning coordinator (NdJ) was not involved in delivering the pedagogic content of the module, but taught both students and staff how to use the software such as ‘Surfgroepen’ and Skype used in its delivery prior to its start. She was also involved in the provision of advice to the module teaching team regarding the adaptation of PBL activities for the online learning environment, and the kinds of equipment students needed to undertake the online module variant in their own home. This included advice to students on optimum internet speeds, microphone and camera equipment etc., and the downloading of the requisite software on their home computers. She also undertook an online test of both the equipment and the software with each student prior to the start of the module and provided technical support to both students and members of the teaching staff (lecturers, PBL tutor etc.) throughout the pilot. She facilitated the recording of lectures during the classroom-based variant and uploaded these onto the requisite pages of the University’s online learning platform ELeUM so that students undertaking the online variant could access them as quickly as possible, and procured additional equipment such as drawing equipment and software necessary for the PBL tutor to illustrate her feedback to students in the online module variant. She also maintained regular contact with the module coordinator and PBL tutor by telephone and email throughout the module, and made sure that she had an online presence during the online tutorials in order to answer any queries which students or the PBL tutor raised in relation to the technical delivery of the module. Her role was restricted to the online module variant where she contributed to the important ‘human reaction’ aspect of the module (Sitzmann et al. 2006).

## Methods

Whilst research suggests that problem-based learning can be successfully applied in online learning environments (Woltering et al. 2009), the approach must be student rather than technology centered (Sitzmann et al. 2006), and the aforementioned principles which guide problem-based learning should be clearly recognisable in the online problem-based variant of such courses. The delivery of a problem-based statistics module using both online and classroom-based variants provided opportunity to compare the relative effectiveness of the two, thus a study was developed to compare the two variants in order to evaluate both the acceptability and success of the online variant.

### Research questions

The Faculty of Health, Medicine and Life Sciences at Maastricht University is actively exploring the use of online and blended learning approaches in combination with problem-based learning for healthcare employees undertaking part-time postgraduate study. This means that students working within the healthcare sector can participate in a combination of classroom and online learning activities depending upon their individual needs. A number of e-learning interventions have been tested elsewhere in relation to the provision of statistics training (Krause et al. 2009), but the primary aim of this study was to implement and evaluate the key learning principles of problem-based learning in an online learning environment and elicit the experiences of all those involved in the process, including the module coordinator, the PBL group tutor, and students undertaking the online variant of our own statistics training in order to make recommendations for future practice. The following research questions were therefore developed for the study:

- Is the online statistics module an acceptable alternative for all stakeholders i.e.
  - Are students satisfied with the online variant of the module?
  - Are the module coordinator, the PBL tutor and the blended learning coordinator satisfied with the online variant of the module?
- Do students undertaking the online variant achieve the learning outcomes for the module to the same extent as those doing the classroom-based module?
- What proposals could be made for the development of further online module variants on the basis of the pilot study?

Ethical approval was obtained from the Faculty of Health Medicine and Life Sciences at Maastricht University. Student participation was entirely voluntary, although in the event, all of the part-time students expressed their willingness to participate in the online learning variant in preference to attending the face-to-face variant on another day of the week. Students in both module variants provided written consent for their participation in the study and there was no attrition from either module variant.

### Research design

The study can be conceptualised as ‘real world research’ (Robson 2002). This means that the research is conducted in a real-life setting in response to the oftentimes multiple and complex contingencies encountered by the practitioner-researcher in the context of their normal professional activities. Rather than trying to control for these variables, the situation is characterised, not to test hypotheses, but to examine the many different aspects that illustrate the phenomena or phenomenon under study using both qualitative and quantitative data (Dolmans et al. 2005). In this sense, it is also ‘developmental research’ (Van den Akker 1999) in that critical elements of online and problem-based learning were identified in relation to an identified need or problem, and used to develop a solution to that problem (i.e. the inability of part-time students to attend the new PBL-based statistics module due to work and other timetabling constraints). This was then implemented in practice, and its outcomes evaluated through a structured form of inquiry i.e. an unmatched two-cohort case-study. This approach highlights the polycontextuality of both educational practice and research (Engeström et al. 1995) and is commensurate with the ‘boundary-crossing’ activities of the ‘researcher as practitioner’ and the ‘practitioner as researcher’ which is inherent in the conduct of research within one’s immediate field of practice (Engeström 2001). Case-study is thus a useful form of research in such circumstances given that they can be used, ‘to investigate a contemporary phenomenon within its real-life context, especially when the boundaries between phenomenon and context are not clearly evident’ (Yin 2003, p. 13).

### Data collection and instruments

In line with the comparative case-study approach used for the inquiry, multiple data sources were used for the purposes of collecting, analysing and interpreting our observations (Yin 2003). These are now discussed, starting with the collection of student-reported data which were collected at two different time points during the study as follows:

A *paper questionnaire* (T1) was developed by the researchers to elicit demographic characteristics, the level of English and the computer literacy of each student in the study. These were rated on a 5-point Likert scale with room for free text comments. There was also an open question asking students to outline what motivated them to undertake the MSc programme. The questionnaire was administered on the first day of the module when both sets of students were present in person at the University.

An *online questionnaire* (T2) was also developed. For students this contained questions regarding the content of the module, the acceptability of the online variant (only online learning students), video lectures (only online learning students), and the quality and usefulness of the tutor feedback. Each of these were rated on a 5-point Likert scale. The online questionnaire also included an instrument developed by Dolmans et al. (1998) to elicit four motivational dimensions (motivation; cohesion; sponging and withdrawing), and two dimensions based on cognitive theories (interaction and elaboration). The aim of this instrument was to expand the team’s understanding of motivational and cognitive influences on tutorial group processes. This instrument consists of 13 statements on a five-point Likert scale. In addition, a mark on a scale from 1 to 10 for the overall tutorial group productivity had to be given (Dolmans et al. 1998). Students completed the questionnaire after finishing the module in its entirety.

#### Use of discussion boards

Active student participation and parity of effort were assessed by monitoring the use of the weekly discussion boards in the online variant. The number of messages delivered, the number of messages read, and the number of agreements were counted and compared to the number and identity of students responsible for the weekly task whose contributions were also evaluated in relation to the student effort suggested by each piece of work.

#### Summative examination results and online participation in the distance-leaning variant

The summative assessment for the module consisted of an unseen multiple choice examination consisting of 25 questions which was taken in the final week of the module (week 8) on university premises. It was hypothesized that the results of these would provide an objective measure of student performance. In addition to this, it was hoped that observation of student contributions to the discussion board together with the frequency with which online resources were accessed and students’ reacted to others’ work would provide an indicative proxy measure of individual student effort for the module. It was acknowledged that the recording of face-to-face tutorials in the classroom-based variant would not be feasible on this occasion, although it has been used in relation to other modules thus investigated.

#### Interviews with the module coordinator and PBL tutor

The module coordinator was interviewed once at the end of the module by a researcher unconnected with the delivery of the module or its development (SO’C). The interview concerned the module coordinator’s experiences of organising the module in the online variant, their time investment, the support of the blended learning coordinator, and the issues related to the development of the online module variant. The PBL tutor was also interviewed by the same researcher after the module ended to elicit her opinions and experiences concerning the module, and the functioning of both the face-to-face and online variants, both of which she was responsible for. The interview also included questions about the level of support required and provided by the blended learning coordinator. Both interviews were audio recorded and supplementary notes were also made. These were summarised by the interviewer and sent to both interviewees so that they could validate the transcripts and make any necessary adjustments, corrections or post hoc comments. The interviewer was an experienced qualitative researcher with a PhD in Educational Research who was also experienced in the delivery of online PBL modules delivered elsewhere within the Faculty.

### Data analysis

Quantitative data were subjected to simple descriptive analyses such as frequencies, means and standard deviations. No further statistical analyses were conducted given the recommended maximum size of no more than 12 students per tutorial group for PBL activities. Moreover, the purpose of the case-study was not to determine statistical significance or extrapolate the findings to other settings, but to determine from a pragmatic, real world perspective whether the strategy of developing and delivering an online variant of the module had succeeded in its primary objective, and whether it could be improved upon. In this respect, and in line with standard case-study practice, traditional notions of sampling logic—and with them, sample sizes were rejected, since it is the quality and appropriateness of observations and not their number which determine the rigour of case-study research (Yin 2003). Qualitative data were subjected to narrative analysis in order to facilitate the telling of the informants’ stories and relaying their experiences of developing and delivering the online problem-based variant of the module. This too is in line with standard case-study objectives which aim to illuminate a decision or set of decisions, why they were taken, how they were implemented and with what result (Schramm 1971).

## Results

In this section the results of the student questionnaires, the use of discussion boards, and the assessment data are first discussed. Qualitative data obtained from interviews with the module coordinator and PBL tutor have also been integrated into the section where appropriate.

### Demographic characteristics of the students and their motivation in doing the module

All students were living in the Netherlands at the time of the study although 2 students in the online variant and two students in the classroom-based variant came from other countries (Ukraine and Germany versus Canada and Germany, respectively). Seventeen students were female, and females predominated in each group. The average age of the online and classroom-based groups was 25.1 years (SD = 1.9) and 26.5 years (SD = 7.9) respectively. Twelve students had a Bachelor’s degree from a Dutch (or Dutch equivalent of a) vocational college (Hogeschool) and eleven students had obtained their degree from a traditional university of higher educational institution. Of these, six were enrolled on the online learning variant and five in the classroom-based variant of the module. There was therefore, a nominal degree of similarity between the two groups in relation to their age, national differences and educational backgrounds. It was noticeable, however, that motivational factors in the online learning group were different from those of the classroom-based group in spite of these apparent similarities. Part-time students said they were studying to improve their professional practice and/or develop their careers, whereas full-time students said that they were doing the module to complete their education, obtain a Master’s degree or improve their knowledge. Only four students within the online variant had prior experience of online teaching or e-learning methods, but this did not seem to affect the work processes within this group. English language seemed not to be a problem, and computer skills were rated by students in both groups as being average to good which is not unusual for postgraduate courses taught within the university.

### Satisfaction with preparation for online learning

Frequent contact between the course and blended learning coordinators to discuss how to organise the course and manage the learning process prior to the course was considered important. The module coordinator mentioned that the preparation given to teaching staff to prepare them for online learning was a little under an hour, but useful and adequate. In common with most of the module teaching team, the PBL tutor responsible for the two groups in the study did not have prior experience of e-learning technologies. She initially disliked the idea of *‘talking into the machine’* and not having immediate interaction with the students as she felt that non-verbal communication and close pedagogic proximity to the students is an important part of the teaching process. She reported that preparation for each session took a little longer as one had to check that camera and microphone settings etc. were working adequately. The help and advice provided by the blended learning coordinator proved useful in this respect and she felt that this was both useful and timely in preparing for the online module. Students too reported that they felt well prepared for online learning during the face-to-face contact with the module teaching staff and the blended learning coordinator during the first taught (face-to-face) day of the module. The preparatory ‘how to’ lecture and discussion session about online learning was at the end of a very busy first day however, and the module coordinator observed that it may have been better to provide this content earlier in the day or prior to the module actually starting. Table 1 demonstrates that students in the online variant of the module found the course to be more interesting with a mean score of 4.0 compared to 3.3, perceived themselves to have learned more (4.2 vs. 3.7), and were more content with the course (4.2 vs. 3.2). They also felt that feedback from the PBL tutor was more relevant, clear and adequate than full-time students undertaking the face-to-face variant. The only item scored lower by online students was satisfaction with the timing of tutor feedback although of course, this was asynchronous in nature (mean or 3.0 compared to 3.4). Thus for most outcomes, students undertaking the online variant seemed more context with the module they were undertaking than those taking the full-time classroom-based variant.

**Table 1:** Comparison of student questionnaires

	Classroom variant			Online variant		
	Mean*	SD	n	Mean	SD	n
*Questions regarding the module*						
The content was interesting	3.3	1.2	9	4.0	0.6	6
The quality of the course was good	3.4	1.2	9	3.8	0.4	6
Overall, I am content about the course	3.2	1.3	9	4.2	0.4	6
I learnt a lot	3.7	1.2	9	4.2	0.4	6
*The tutor feedback was*						
Relevant	3.9	0.8	9	4.0	0.6	6
Clear	4.0	0.5	9	4.2	0.4	6
Sufficient	3.9	0.8	9	4.2	0.4	6
Timely	3.4	1.2	9	3.0	0.9	6
*Motivational and cognitive processes*						
Motivational						
Motivation	2.7	0.8	9	3.6	0.9	6
Cohesion	2.9	0.9	9	3.1	0.8	6
Sponging	3.4	0.9	9	3.1	0.9	6
Withdrawing	2.9	0.8	9	3.1	0.7	6
Cognitive						
Interaction	2.8	0.7	9	3.1	0.8	6
Elaboration	3.1	0.8	9	3.4	0.7	6

* Range is 1–5 (completely disagree—agree completely)

### Technical support during the module

The module coordinator stated that the level of technical support provided by the blended learning coordinator was excellent. There was no evidence that students were having difficulties using the technology and responses were always prompt and adequate. Students also reported that their comments/issues were dealt with very quickly.

### Course and variant

Seven students in the face-to-face group spent 11–20 h per week on the course. The other two students spent less time on their studies. All students in the online group spent 10 h or less on the course. All students considered the variant they followed to be acceptable. The online learners stated that they would choose the same variant again. The freedom provided by these variants was appreciated. In an open question, students in the online variant mentioned that the format could be easily used for other courses, one student for example writing, ‘please continue to develop these kinds of courses and actively offer them to alumni of the university. I miss these kinds of courses as an alumni’. Another student said, ‘the way this course was organized was very good. I would support more of these courses’, whilst a third said, ‘I would choose this form of education again for a statistics course. For another course it would also be applicable’.

### Video lectures

The students in the online variant rated the quality of video lectures as good and said that these were delivered in time. The module coordinator reported that some of the lectures filmed in the classrooms were not ideal. The lecturer was visible, but the PowerPoint slides were not always visible. Unless the students had printed the handouts in advance, they could not see important diagrams or equations. The module coordinator emphasised that for a subject such as statistics, students must be able to see the slides as well as the lecturer. The module coordinator also mentioned that on a couple of occasions, the audio equipment was not working in the teaching room where lectures were being recorded. This posed a problem for the lecturers, but did not deleteriously affect recording for online learning purposes since a separate microphone was used to record these.

### Tutoring

One student said, ‘I think that personal meetings [with the tutor] are also recommended. Maybe every two weeks or something to discuss problems etc*.,* but on the whole, students in the online group seemed to have little difficulty with the fact that no tutor was involved during the asynchronous online tutorials. All but one of the students in this group said that it was possible to send questions to the tutor by email etc. which were answered promptly. In contrast, the face-to-face group said that they would not choose a variant without a tutor, arguing that this was a necessary component of the course for them. Feedback from the tutor was relevant, clear and sufficient for both groups. Mean scores on the study questionnaire were 3.9 or higher on a scale of 1–5 although the online group felt that feedback was not always timely (mean 3.0).

The PBL tutor referred to the situation in which she gave tailored feedback on the summary of the online learning group saying, ‘The blended learning coordinator was always present and it was nice to know she was nearby to help out if needed’. When audio-taping feedback on the written reports, the tutor found that her responses were not as ‘natural’ as they might be in a face to face meeting. She had to think longer and harder about what response to give, and how to portray this to the students on the audio voiceover. She sometimes found it hard to understand specifically what the students were saying and ‘had to guess’ what they meant at times. She regarded this as less optimal than the face-to-face group as there was no opportunity to clarify questions with the students. The tutor was also frustrated at the poor visual acuity of the ‘pen’ of the Wacom tablet used to draw and highlight diagrams when annotating feedback. The visual representation on the computer screen was often less accurate than she was drawing, and she felt that it looked as if she had poor psychomotor coordination, highlighting the importance of having the best quality of technical equipment for such purposes .

### Use of discussion boards in the online variant

The discussion board ‘questions to the blended learning coordinator’ was used four times. On three occasions, a technical question was asked and the fourth concerned the availability of a recorded lecture on ELeUM. The following discussion boards were not used at all: ‘questions to the tutor’ and the ‘coffee corner’. On the other hand, discussion boards regarding specific statistical problems were visited regularly. The majority of students were responsible for two problems during the course. Half the students posted less than ten messages on the discussion boards in respect of these. Two of these students displayed a relatively high number of agreements and three were responsible for one problem at a time. No-one in the online group read all of the messages (156 messages) posted by their colleagues and it was noticeable that seven participated more frequently than others. These seven delivered comments and registered their agreement or disagreement 23 times or more.

The tutor did not have to manage the discussion boards on Blackboard at all since the blended learning coordinator took responsibility for monitoring its use. The tutor liked the fact that the blended learning coordinator was monitoring the students regularly to remind them of their tasks and duties. The tutor mentioned that it would be too time consuming for her to do that, but found it very important. Constant reminders of the tasks which needed completion certainly played a role in students’ commitment and individual performance in the course.

### Motivational and cognitive influences on tutorial group processes

Motivation considers whether the tutorial group stimulated their own self-study activities and whether these had a positive effect on their effort and commitment. The face-to-face group scored relatively low in this domain whereas part-time students were more positive about the value of this. The part-time students also scored more highly on cohesion, i.e. the students felt they were members of a distinct group with clearly defined responsibilities. Students in both groups indicated that some group members had a negative effect on the commitment/efforts of other group members in respect of their ‘sponging’ from others’ efforts. The dimension ‘withdrawing’ consists of two items: (1) During the flow of the course, some group members contributed less to tutorial group discussions and, (2) some group members intentionally withheld information they had acquired during self-study. The online learning group had a higher score on this dimension compared to the face-to-face group, indicating that they were less likely to share significant information with other group members. The motivational and cognitive dimensions measured by the instrument of Dolmans et al. (1998) are also described in Table 1. This shows a mean score for motivation of 3.6 in the online group in comparison to 2.7 for the classroom-based variant. Interestingly, they also scored more highly for group cohesion and not withdrawing from the learning activity (3.1 vs. 2.9 in each case). Sponging behaviours (i.e. passive involvement in group activities without making a significant contribution) were lower in the online variant of the module (3.1 compared to 3.4), and both student interaction and their willingness to elaborate on personal learning were higher in the online variant (3.1 and 3.4 respectively for online students vs. 2.8 and 3.1 for each of these variables respectively).

In relation to the cognitive dimension, part-time, online students reported more interaction and elaboration in their tutorless group meetings compared to the face-to-face group. Within the interaction dimension, students stated that they had learned much more from the contribution of others, encouraged each other to critically discuss the subject matter in more detail, and had corrected each others’ misconceptions during group interactions. Elaboration was defined as the provision of explanations to each other and providing explanations in their own words. Marks for group productivity (range: 1–10) in the face-to-face and online groups was 6.9 and 7.0 respectively. Although group productivity is not highly rated in either group, a certain extent of collaboration should exist within the course.

### Face-to-face group versus the online according to the PBL tutor

The tutor mentioned that the online students were generally older and clearly trying to ‘make something more out of their careers’. She said: ‘I was very surprised with the level of commitment and they seemed to be very good’. There was also *‘greater maturity’* in the quality of the discussions which were very good in both online groups in comparison to the face-to-face group. She felt that the face-to-face group were *‘less active’* and *‘less involved’* in the learning process as it was easier for the tutor to ‘step in’ in response to students’ non-verbal communication to address areas in which they seemed less certain about the content. Nevertheless, the online learning group seemed to be particularly independent in their learning in comparison to the face-to-face group.

### Examination results

Twenty-one students passed and two students failed the unit test (one from the online and one from the face-to-face variants). The average mark for the unit test was 7.3 (SD = 1.3; min–max: 4–9) in the face-to-face group and 7.8 (SD = 1.5 min–max: 4–10) in the online learning group, an insignificant finding indicating that there was no meaningful difference in examination outcome between the two groups.

## Discussion

The aim of the study was to implement and evaluate the key learning principles of problem-based learning within an online statistics module. It was apparent from the study that both groups of students were engaged in the active construction and reconstruction of learning. One principle underlining problem-based learning is that learning is a socially constructed activity which involves the active collaboration and participation of all learning group members in response to problem learning statements (Hmelo-Silver and Derry 2008). We found no evidence that this method had a deleterious effect on the students in this pilot—at least when measured against those undertaking traditional face-to-face, synchronous problem-based learning. Cook (2007) argues that synchronous online discussions are similar to small-group face-to-face sessions since teachers can still facilitate group learning in a manner which is likely to be equally effective as synchronous discussions. In our study, students doing the online variant said that they did not feel the need for synchronous facilitation of their discussions by a tutor, and it is likely that the advantage of not having to travel or meet together at a prearranged time suited to meet the tutors availability outweighed any disadvantages experienced in not having immediate and direct contact with a tutor. Moreover, it is possible that the absence of a formal group tutor made students rely more heavily upon each other and develop their own critical thinking, analytical and self-regulation skills as discussed above (Gray and Tobin 2010; Barnard et al. 2009; Goodrich 2007).

In contrast to this, both the module coordinator and the PBL tutor said that they would have preferred the discussions to have been conducted synchronously in the presence of the tutor and there is certainly some evidence that higher levels of online interaction between the PBL tutor and the students may have resulted in even greater levels of motivation within this group (Sitzmann et al. 2006; Brown and Ford 2002; Entwistle and Entwistle 1991). However, in contrast to the face-to-face variant, online discussions may have been more advantageous in allowing students time to think about the issues before responding to them, and constructing a more thoughtful response within the complicated ‘turn-taking’ interaction necessitated by online learning environments (Cook 2007). This may account for the PBL tutor’s perception that the online learning students were more mature in their approach to learning, but it is necessary to consider other factors too before drawing a final conclusion.

### Did all of the students attain the required learning results?

The need for objective measures of educational effectiveness as well as perceptions, attitudes, self-ratings and opinions has been forcefully argued within the problem-based learning literature (Colliver and Markwell 2007) so it is appropriate to begin this discussion with the objective measure of student examination outcomes as a proxy for the pedagogic effectiveness of the online learning variant. All but one of the students undertaking this passed the examination. Notwithstanding this, the mean score for students undertaking the online variant was slightly better than those taking the face-to-face variant in spite of their not having face-to-face tutorials with a statistics lecturer. The problem-based learning tutor (who supervised both groups) felt that students in the online variant were generally more committed to their learning and showed greater maturity than the full-time students undertaking the face-to-face variant. The ability of students undertaking online modules to successfully self-regulate themselves in this way has been reported elsewhere within the literature, Barnard et al. (2009) for instance, noting that it may also be associated with increases in overall academic achievement. It would appear therefore, that online learning was useful not merely used as a means for transmitting information, but helping students to engage in flexible, learner-centred teaching which encouraged greater interaction amongst those undertaking the blended learning variant (Ellaway and Masters 2008).

These differences suggest that the more expansive or horizontal view of education in which the primary object of learning is to transform professional activity may also have played a part in their commitment to their studies (Engeström and Sannino 2010) although this cannot be said with any degree of certainty. It was noticeable that the student failing the module test in the online variant had participated much less in the online learning activities, and this again bears out Barnard et al.’s findings that in order to work, students must display a high level of personal motivation and self-regulation in order to take advantage of the learning opportunities afforded by online learning The absence of material artifacts such as the recorded discussion board for the face-to-face variant make it much harder to determine likely reasons for the failure within this group or objectively identify those student inclined to procrastination or lack of commitment to their course work which may be one advantage of the blended learning variant (Romano et al. 2005).

### Were students satisfied with the online statistics module?

Less than half the online learning group were familiar with e-learning at the outset of the study, but this did not seem to affect the work processes or social cohesion of the group by the end of the study. It is particularly interesting that students doing the online variant scored higher than the face-to-face group on group cohesion given that they only met once on the first day of the module and did not meet again in person until the module examination in the final week of the module. It is hoped that the very determined attempts of the module team to instil a sense of teamwork amongst students both in the initial face-to-face meeting and subsequent online sessions was an important consideration in the development of a successful ‘online community’ (Gray and Tobin 2010) or the establishment of a strong ‘cognitive presence’ (Vaughan and Garrison 2005), although the students’ experiences of working collaboratively with others in a variety of healthcare settings may also have played a part in this encouraging though tentative outcome. It is also possible that these students were better prepared for this important ‘boundary-crossing’ activity during the module by virtue of their also occupying a world of work in which team-working and flexibility are all important (Engeström 2001).

### How satisfied were the module teaching staff with the online variant?

Data from the student questionnaires indicate that participants in the online variant were more positive about the module and the role of the personal tutor than those doing the face-to-face variant, a finding consistent with the findings from Woltering et al.’s (2009) mixed-method evaluation of 185 medical students undertaking an online problem-based learning course in Aachen, Germany. Students appeared to appreciate the high degree or freedom afforded them to decide when and how to study the module content in response to the weekly learning tasks. Each said that they would choose the same variant again, and it is interesting that they did not regard themselves as having been disadvantaged by the absence of a tutor from their deliberations. On the contrary, both she and they felt that individual members had taken the initiative and managed the process satisfactorily without this oversight, indicating once again the maturity of this group of students (Barnard et al. 2009). This subjective impression seems to be supported by the more objective group scores for interaction, elaboration and group activity in the online questionnaires, although analysis of student participation in the weekly learning activities still showed individual variation in the use of the student discussion boards as previously discussed. The same is likely to be true of the face-to-face tutorials however, although the absence of any lasting artifacts from these group discussions makes it harder to establish the extent of this variance and one has to rely upon student self-reports as to the scope and scale of their contribution to group learning activities. Students in both groups commented upon the negative effect which ‘free-riders’ had upon group dynamics and learning attainment, but the problem appeared to be no greater in the untutored online variant of the module that the traditional face-to-face classroom variant.

Interview data show that both the module coordinator and problem-based tutor were initially sceptical about the potential of online learning to deliver a complex statistics module, findings which are consistent with much of the blended learning literature (Turney et al. 2009; Romano et al. 2005). Ultimately however, they found it to be an enjoyable and acceptable alternative to traditional classroom teaching, and this highlights the importance of gaining an adequate level of ‘buy-in’ from both teachers and course planners about the use of online learning technologies for higher education purposes (Lahaie 2007). The role of the blended learning coordinator also appears to have been important in this respect, since reassurance about the potentialities of web-based learning technologies seems crucial to their successful uptake and their role appears to have been quite unique in this respect (Lahaie 2007; Covington et al. 2005). Both the problem-based learning tutor and the module coordinator found that the delivery of the online variant did not take significantly more time than the face-to-face variant, except for that spent providing written rather than immediate verbal feedback in response to student questions or the problem-based tasks themselves. The support of the blended learning coordinator was considered important in this respect however, since she took responsibility for much of the technical and procedural activities, including reminding students about deadlines and alerting them to the availability of new learning resources on the module’s ELeUM (Blackboard) site. The ability to monitor what students were actually doing and who was contributing most to the learning tasks was regarded as an advantage over the face-to-face variant since group interactions or individual contributions made outside the classroom environment were largely ‘invisible’ to teaching staff in the face-to-face variant.

### What changes should be proposed on the basis of particpants’ experiences?

Having evaluated the conduct of the module it is apparent that small improvements of a technical or organisational nature could be made in the future and this is wholly appropriate since the quality of the approaches used in teaching and learning are undoubtedly related to the quality of learning outcomes (Ginns and Ellis 2007). Future changes would include amendments to the scheduling of the module introduction so that more time is spent demonstrating the use of the learning technologies to be used to both students and members of the teaching staff. They would also include the more timely provision of video recorded lectures and student handouts on Blackboard so that students could annotate these during the pre-recorded video-lectures. Improvements could also be made to the visual and auditory acuity of video recorded lectures which should be of the best possible quality and present both an image of the speaker and the visual illustrations (PowerPoint slides etc.). Similarly, investment should (and has been) directed towards the purchase of the best possible quality of ‘drawing tool’ used for capturing complex visual illustrations in digital format to illustrate the answers to student questions. Both the problem-based learning tutor and the module coordinator indicated that they would prefer to provide synchronous feedback to students, although students indicated that they were generally satisfied with the quality and level of feedback given via the audiovisual recordings.

### A future role for the blended learning coordinator?

The blended learning coordinator was heavily involved in the technical delivery of the online variant in this pilot project and in motivating students to complete weekly learning tasks. In future versions of the module, it is assumed that much of this work will be undertaken by the problem-based learning tutors themselves with much less ‘hands-on’ involvement on the part of a single blended learning coordinator. It is therefore advisable that the role is clearly defined prior to any such venture in the future, and all involved in setting up similar projects to consider the academic quality standards required for e-learning and teaching (Kennedy 2005; Ginns and Ellis 2007). In addition, the development of an online assessment strategy for the conduct of the individual knowledge test would also assist students travelling from long distances to avoid the time and travel costs involved in taking a classroom-based ‘pen and paper’ examination and there is no reason why the conduct of this test cannot also be supervised remotely by the relevant academic staff.

### Limitations of the study

Some of the results indicated by this study may be influenced by the lack of homogeneity between full-time, face-to-face students and those undertaking the part-time, online variant of the module as part of their Masters programme. Attempts to distribute full-time and part-time students across both variants would have obviated the purpose of the online delivery however, since this was specifically developed to overcome timetabling issues and the demands placed by the course upon busy working professionals who could not undertake two study days per week. The online variant seemed to be particularly suited to these students however, in contrast to full-time students who said that they would prefer to receive face-to-face tuition. It is hard to compare the different groups directly therefore, particularly since two students did not complete the paper and pencil questionnaire and eight students did not respond to the online survey at the end of the module. It is recognised that pen and paper surveys elicit a higher response rate among students than online surveys do (Sax et al. 2003), which is why further statistical tests have not been employed on the data.

## Conclusions

The freedom provided by the provision of an asynchronous online learning variant of a statistics module on factor analysis and the multilevel analysis of longitudinal data was highly appreciated by students whose significant work-life responsibilities prevented them from taking a classroom-based face-to-face module. Use of a previously validated online questionnaire suggested that the ‘self-directed learning’ principles which underlie problem-based learning within the study were clearly met within this exploratory module variant, and these findings were objectively correlated by observation of both individual and group learning activity on the Blackboard based e-learning system used to deliver the module content and facilitate group learning activities. The online learning variant enabled the students to successfully manage their continuing professional development more effectively without the need for additional time off work, without affecting the quality of their learning experience as measured by their summative module test results and the perceptions of both the group tutor and the module coordinator responsible for students undertaking both variants of a module. These contained exactly the same module content and was intended to meet identical learning outcomes. Whilst the results of this small exploratory case-study cannot be generalised to other curricula or educational settings, it appears at least tentatively, that a relatively complex academic subject can be successfully taught using a problem-based online format, raising the possibility that students living some distance from the university—or even abroad might be able to participate in such a module in future and successfully meet both the aims and objectives of problem-based learning, and the content specific learning outcomes for the module.
